# *E. coli* ST11 (O157:H7) does not encode a functional AcrF efflux pump

**DOI:** 10.1099/mic.0.001324

**Published:** 2023-04-19

**Authors:** Hannah L. Pugh, Christopher Connor, Pauline Siasat, Alan McNally, Jessica M. A. Blair

**Affiliations:** ^1^​ Institute of Microbiology and Infection, College of Medical and Dental Sciences, University of Birmingham, Edgbaston, Birmingham, B15 2TT, UK; ^2^​ The Francis Crick Institute, 1 Midland Road, London, NW1 1AT, UK; ^3^​ Department of Microbiology and Immunology at the Peter Doherty Institute for Infection and Immunity, University of Melbourne, Melbourne, Victoria 3010, Australia

**Keywords:** *Escherichia coli*, RND, efflux, ST11, *acrF*

## Abstract

*
Escherichia coli* is a facultative anaerobe found in a wide range of environments. Commonly described as the laboratory workhorse, *E. coli* is one of the best characterized bacterial species to date, however much of our understanding comes from studies involving the laboratory strain *E. coli* K-12. Resistance-nodulation-division efflux pumps are found in Gram-negative bacteria and can export a diverse range of substrates, including antibiotics. *E. coli* K-12 has six RND pumps; AcrB, AcrD, AcrF, CusA, MdtBC and MdtF, and it is frequently reported that all *E. coli* strains possess these six pumps. However, this is not true of *E. coli* ST11, a lineage of *E. coli *, which is primarily composed of the highly virulent important human pathogen, *E. coli* O157:H7. Here we show that *acrF* is absent from the pangenome of ST11 and that this lineage of *E. coli* has a highly conserved insertion within the *acrF* gene, which when translated encodes 13 amino acids and two stop codons. This insertion was found to be present in 97.59 % of 1787 ST11 genome assemblies. Non-function of AcrF in ST11 was confirmed in the laboratory as complementation with *acrF* from ST11 was unable to restore AcrF function in *E. coli* K-12 substr. MG1655 Δ*acrB* Δ*acrF*. This shows that the complement of RND efflux pumps present in laboratory bacterial strains may not reflect the situation in virulent strains of bacterial pathogens.

## Data Summary

Supplementary tables and figures are found at the end of this manuscript. The pangenome dataset is available at 10.6084 /m9.figshare.c.6147189 and the 1787 ST11 genome assemblies are available at 10.6084 /m9.figshare.21975647.

**Impact Statement**

RND pumps are involved in a wide range of physiological functions. However, it is their role in antimicrobial resistance and bacterial virulence that are of significant clinical interest. Still, most of what we know is based upon data from single representative type strains. Here we demonstrate that the study of single type strains results in genetic diversity being missed.

## Introduction

*Escherichia coli* is a Gram-negative bacterium found in a diverse range of environments, including soil, water and both the human and avian digestive tracks. The first published *E. coli* genome was of the well-studied laboratory strain, *E. coli* K-12 substrain MG1655 [1]. K-12 is commonly taken as a representative of the *E. coli* species, however the first genome sequence of a pathogenic *E. coli* strain, *E. coli* O157:H7, demonstrated wide genetic diversity and genome size variation [1, 2].

As genome sequencing has become more accessible, several methods for classifying *E. coli* assemblies have been defined. *E. coli* assemblies can be split into seven main phylogroups (A, B1, B2, D, E, F, G), which are groups of genomes clustered by phylogenetic similarity [3]. Isolates can also be typed by multilocus sequence typing (MLST), a method based upon the sequence of seven housekeeping genes. For example, using the Warwick MLST scheme, *E. coli* O157:H7, which is a serological typing, is classified as sequence type (ST) ST11. Interestingly genome size has been shown to vary not only between ST but trends are also seen between the different phylogroups, with niche-specific bacteria having much smaller genome sizes than those that live in the environment [4, 5]. Furthermore pangenome size has also been shown to vary between ST groups [6]; pangenome referring to all genes within the given population.

Resistance-nodulation-division (RND) efflux pumps are tripartite complexes found in Gram-negative bacteria. Many have broad substrate ranges, which include antimicrobials, dyes, detergents and solvents [7], however some, such as CusABC, which exports copper and silver ions, have very narrow substrate ranges. The best characterized RND system is AcrAB-TolC, homologs of which are found in *E. coli, Salmonella* and *Klebsiella *. Antimicrobial resistance can occur via this system due to mutations in regulators resulting in increased expression or mutations within the pump itself leading to the modification of substrate specificity [8–10]. Furthermore, RND efflux pumps have been shown to be involved in virulence across Gram-negative bacteria, including but not limited to *Klebsiella pneumoniae, Salmonella enterica, Enterobacter cloacae* and *Erwinia amylovora* [11–14]. The RND pump AcrF is a close homolog of the major pump AcrB; in *E. coli* the AcrF protein shares 77  % identity with AcrB, and 84  % nucleotide identity [15]. Yet unlike *acrAB*, *acrEF* is not expressed constitutively under laboratory conditions due to HNS silencing [16–18].

As mentioned, *E. coli* O157:H7 (ST11), is a frequent foodborne human pathogen though its primary reservoir is the ruminant digestive tract [19]. Disease in humans is caused through the secretion of the Shiga-toxin (Stx), which following host cell uptake is cleaved into two subunits, with subunit A targeting the ribosome resulting in protein synthesis inhibition and cellular apoptosis [20, 21]. Stx-producing *E. coli* infections can be asymptomatic, however symptoms of disease can vary from watery diarrhoea to haemolytic uraemic syndrome (HUS) and subsequently kidney failure [22]. Despite changes in UK hygiene practices, the number of *E. coli* O157 infections annually between 1983 and 2012 remained consistent, with an average of 887 cases a year [23].

In this work, the conservation of both the AcrAB and AcrEF efflux systems, plus their associated outer membrane protein (OMP), TolC, was determined across the pangenomes of 18 *E. coli* ST. The absence of AcrF across the ST11 lineage was confirmed at the individual assembly level and the conserved protein sequence was proven to be non-functional *in vivo*.

## Methods

### Pangenome construction

Pangenome construction is described in [24]. Briefly, assemblies were downloaded from the Enterobase *E. coli/Shigella* database [25]. Duplicate assemblies were removed from the analysis using mash (v1.1.1) [26] prior to annotation using prokka (v1.12) [27]. Pangenomes for each ST were then constructed using Roary (v3.10.2) using the default parameters; core-gene alignments were produced using mafft [28]. Table 1 lists all STs used and the number of assemblies included in the analysis. Assemblies are available at 10.6084 /m9.figshare.c.6147189. The STs selected were chosen to span the main phylogroups of *E. coli* as well as multiple pathotypes and commensal lineages. Extraintestinal pathogenic *E. coli* (ExPEC) lineages were of particular interest due to their clinical and AMR significance.

**Table 1.** Phylogroups and sequence types included in the analysis

Phylogroup	No. of assemblies	Sequence type
A	2370	ST10
B1	40	ST3
B1	1884	ST17
B1	2441	ST21
B2	283	ST12
B2	62	ST14
B2	46	ST28
B2	873	ST73
B2	758	ST95
B2	232	ST127
B2	3186	ST131
B2	91	ST141
B2	65	ST144
B2	54	ST372
D	696	ST69
E	5137	ST11
F	269	ST117
F	382	ST648

### Identification of RND gene alleles across the pangenome

The gene sequences of *acrA, acrB, acrE, acrF* and *tolC* were downloaded from *E. coli* K-12 substrain MG1655 (accession: NC_000913.3) and aligned using blast (v2.10.0) [29] to the Roary reference genome of each ST pangenome. Sequences with ≥95  % identity to the known sequence from MG1655 were grouped together and deemed to be a single gene ‘allele’. Matches were included in the analysis providing they had an identity percentage ≥95  %, frequency ≥1  % and the expected length for the gene in question (*acrA* 1194 bp, *acrB* 3150 bp, *acrE* 1158 bp, *acrF* 3105 bp, *tolC* 1482 bp). Those that did not exactly meet all three criteria were checked manually to determine whether they were in fact sequences of the gene in question.

### Alignment of *acrF* gene regions

To further investigate the presence and absence of *acrF* in ST11, the region between flanking genes, *acrE* and *yhdV,* was downloaded from 11 ST11 sequences available on NCBI (Table S1, available in the online version of this article). The regions were then aligned using muscle (v3.8.1551) [30] to the *acrF* nucleotide sequence of *E. coli* K-12 substr. MG1655 (accession: NC_000913.3 : 3415033–3418137). Nucleotide sequences were translated to determine the ST11 protein sequence within the region.

### Confirming the *acrF* insertion in over 1700 ST11 assemblies

Additional genome assemblies of ST11 (*n*=1999) were downloaded from the Enterobase *E. coli/Shigella* database and duplicate sequences were removed again using mash (genome assemblies are available at 10.6084/m9.figshare.21975647). These assemblies were then aligned to the 3150 nucleotide *acrF* sequence determined in the above section from *E. coli* O157:H7 str. TW14359 (accession: CP001368.1) using blast.

### Cloning of *acrF* from ST11 into pET21a

The a*crF* gene from *E. coli* K-12 substr. MG1655 and *E. coli* O157:H7 (NCTC 12900) were both amplified from the respective chromosome using Q5 hot start high-fidelity polymerase (NEB) and the following primers; 5′-ttgaccatatgGCAAACTTTTTTATTCGAC and 5′-ggtgctcgagTTATCCTTTAAAGCAACGGC. The primers introduced the *Nde*I and *Xho*I sites, allowing the incorporation of amplimers into the high-copy vector, pET21a (Invitrogen). Both vectors were then transformed into *E. coli* K-12 substr. MG1655, *E. coli* K-12 substr. MG1655 Δ*acrB, E. coli* K-12 substr. MG1655 Δ*acrB* Δ*acrF*.

### Measurement of minimum inhibition concentration (MIC)

The MIC of ethidium bromide, rhodamine 6G and erythromycin were determined by broth microdilution according to the EUCAST guidelines [31]. Strains used in this assay are listed in Table 2. Fold changes greater than twofold were deemed significant.

**Table 2.** Strains used in this study

Strain	Reference
* E *. *coli* ATCC 25922	PHE
* E *. *coli* K-12 substr. MG1655	[1]
* E *. *coli* K-12 substr. MG1655 Δ*acrB*	[43]
* E *. *coli* K-12 substr. MG1655 Δ*acrB* Δ*acrF*	This study
* E *. *coli* K-12 substr. MG1655 Δ*acrB* Δ*acrF*+pET21 a	This study
* E *. *coli* K-12 substr. MG1655 Δ*acrB* Δ*acrF*+pET21 a K-12 *acrF* sequence	This study
* E *. *coli* K-12 substr. MG1655 Δ*acrB* Δ*acrF*+pET21 a ST11 *acrF* sequence	This study

## Results

### Both *acrAB* and *acrEF* are highly conserved across *E. coli* lineages

Pangenomes of 18 ST were constructed from a total of 18 869 genome assemblies and the variation of *acrA, acrB, acrE, acrF* and *tolC* was determined across. In this context, allele refers to a group of sequences with ≥95  % sequence identity to one another. Across the 18 lineages, the mean number of alleles for *acrA, acrB, acrE* and *tolC* was between 1.1 and 1.3 (Table 3). However, *acrF* had a mean of 1.7 alleles per ST (Table 3).

**Table 3.** Alleles of *acrA, acrB, acrE, acrF a*nd *tolC* across the pangenomes of 18 *E. coli* ST Allele conservation for five resistance-nodulation-division (RND) genes across 18 sequence types (ST) of *E. coli *. Here, allele refers to a group of sequences with ≥95 % sequence identity to one another. ST11 had no *acrF* allele.

			Resistance-nodulation-division gene				
Phylogroup	*n*	Lineage	*acrA*	*acrB*	*acrE*	*acrF*	*tolC*
A	2370	ST10	1	1	2	5	2
B1	40	ST3	1	1	1	1	1
B1	1884	ST17	1	1	1	2	1
B1	2441	ST21	1	1	2	1	1
B2	283	ST12	1	2	2	4	1
B2	62	ST14	1	1	1	2	1
B2	46	ST28	1	1	1	1	1
B2	873	ST73	2	2	1	1	1
B2	758	ST95	2	1	1	1	1
B2	232	ST127	2	1	1	1	1
B2	3186	ST131	1	2	2	2	1
B2	91	ST141	1	1	1	1	1
B2	65	ST144	1	1	1	2	1
B2	54	ST372	1	1	1	1	1
D	696	ST69	1	1	2	2	1
E	5137	ST11	2	2	1	0	1
F	269	ST117	1	1	1	2	2
F	382	ST648	1	1	1	2	1
Total	18 869	Mean number of alleles	1.2	1.2	1.3	1.7	1.1

The gene encoding *tolC,* the OMP for five of the six *E. coli* RND systems, was the most conserved across the 18 lineages (Table 3). Both *acrA* and *acrB* were also found to be highly conserved with most STs included in the analysis possessing only a single allele across the respective pangenome (Table 3). The PAP associated with *acrF*, *acrE*, had a mean of 1.3 alleles per lineage. However, the most diverse gene across the dataset was *acrF*. ST10 was found to have five *acrF* alleles across the lineage yet no alleles were present in the ST11 pangenome. Moreover, aligning the 5137 individual assemblies used to construct the ST11 pangenome against the *acrF* gene from MG1655 using blastn did not identify an *acrF* gene, suggesting *acrF* in ST11 isolates is not the same as in *E. coli* K-12.

### The *acrF* gene in ST11 has a conserved 45 bp insertion

**Fig. 1.**
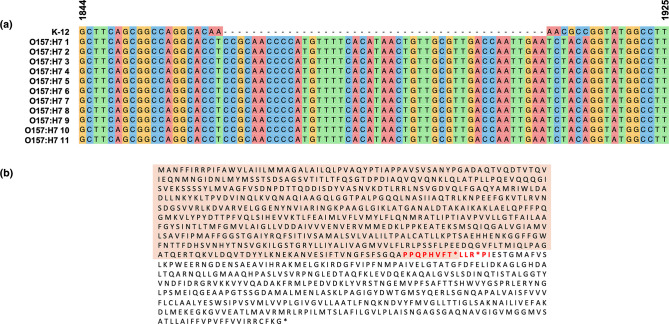
*acrF* in *E. coli* ST11 contains a 45-nucleotide insertion. (**a**) Alignment of 11 ST11 *acrF* sequences downloaded from NCBI database confirms an insertion, not present in *E. coli* K-12 substr. MG1655. (b) Translation of these genes (example O157:H7 2) confirms the insertion contains two internal stop codons.

To confirm the absence of *acrF* across ST11, the region downstream of *acrE* where *acrF* is located was downloaded from 11 *E. coli* O157:H7 genomes available on NCBI as O157:H7 is a very well-characterized ST11 strain. Aligning these sequences using *acrF* from *E. coli* K-12 substr. MG1655 as a reference revealed an insertion of 45 nucleotides between nucleotides 1861 and 1914 (Fig. 1a), extending the *acrF* gene from 3105 to 3150 nucleotides. *In silico* translation of this 45 bp product revealed an insertion of 13 amino acids plus two internal stop codons suggesting a truncated product (Figs. 1b and S1).

To validate this finding 1999 assemblies of *E. coli* ST11 were downloaded from the Enterobase *E. coli/Shigella* database. A total of 212 assemblies were removed from the analysis as they were duplicates. The remaining 1787 assemblies were then used to confirm whether the insertion found in the 11 reference ST11 genomes was conserved in a larger sample size. Using the *acrF* sequence from *E. coli* O157:H7 strain TW14359, an assembly used in the preliminary alignment, it was confirmed that 1744 out of the 1787 assemblies (97.59 %) had a gene that matched the length expected for ST11 (3150 nucleotides). Sequence identity of those matches ranged from 99.56–100  %, with the average percentage identity at 99.94 %, showing that the insertion is highly conserved within ST11 genomes. Of the 43 genome assemblies that did not have the 3150 bp sequence, 7(0.39 %) had the K-12 sequence. In the remaining 36 genome assemblies, no *acrF* gene sequences were identified in alignments against either the K-12 and O157 sequences because either there were additional deletions within *acrF,* the *acrF* gene was entirely absent, or the gene locations on the assembly contigs prevented identification of *acrF*.

### AcrF in *E. coli* ST11 is non-functional

While the presence of two stop codons suggested AcrF was non-functional in ST11, the *acrF* sequences from MG1655 and ST11 NCTC 12900 were each cloned into the high-copy vector pET21a. In order to investigate the function of AcrF, *acrB* was also inactivated as it encodes the major RND efflux pump AcrB, which is known to mask the subtle phenotype of *acrF* expression. The function of AcrF was determined by measuring susceptibility to the known AcrF substrates, ethidium bromide, rhodamine 6G and erythromycin.

For the three substrates tested, deletion of *acrB* significantly reduced the MIC when compared to *E. coli* K-12 subsp. MG1655, but the additional interruption of *acrF* had no additive effect. Complementation with the *acrF* sequence from *E. coli* K-12 did not alter erythromycin susceptibility, however the MICs for ethidium bromide and rhodamine 6G increased from 8 µg ml^−1^ to 64 µg ml^−1^ and 16 µg ml^−1^ to 64 µg ml^−1^, respectively. Complementation with the ST11 sequence had no significant effect on susceptibility for any of the compounds tested confirming that AcrF is non-functional in *E. coli* ST11 (Table 4).

**Table 4.** Susceptibility of *E. coli* K-12 substr. MG1655 Δ*acrB* Δ*acrF* following complementation with *acrF* from *E. coli* K-12 substr. MG1655 and *E. coli* O157:H7 (ST11)

	MIC (µg ml^−1^)		
Strain	ERY	R6G	EtBr
* E. coli* ATCC 25922	64	1024	256
* E. coli* K-12 substr. MG1655	64	512	512
* E. coli* K-12 substr. MG1655 Δ*acrB*	4	8	8
* E. coli* K-12 substr. MG1655 Δ*acrB* Δ*acrF*	4	8	8
* E. coli* K-12 substr. MG1655 Δ*acrB* Δ*acrF*+pET21 a	4	16	8
* E. coli* K-12 substr. MG1655 Δ*acrB* Δ*acrF*+pET21 a K-12 sequence	8	64	64
* E. coli* K-12 substr. MG1655 Δ*acrB* Δ*acrF*+pET21 a ST11 sequence	4	16	8

ERY, erythromycin; EtBr, ethidium bromide; R6G, rhodamine 6G.

## Discussion

Across the Gram-negative bacteria it is generally assumed that efflux pump sequences are conserved across a species, with genes present in common laboratory strains often taken as representatives of a whole species. This is despite several studies demonstrating that clinical isolates of *Acinetobacter baumannii* can lack *adeB,* which encodes the RND component of the AdeABC system associated with reduced antimicrobial susceptibility [32–34]. Here we show that while it is assumed that all *E. coli* isolates possess AcrB, AcrD, AcrF, CusA, MdtBC and MdtF, this is not true for the ST11 lineage.

*E. coli* ST11 is a highly virulent *E. coli* lineage, which includes the Stx producing *Escherichia coli* O157:H7. While the main reservoir of O157:H7 is the recto-anal junction of ruminants [35], it is also a frequent foodborne pathogen in humans causing bloody diarrhoea and in severe cases, HUS through the secretion of Stx [36].

AcrB is regarded as the major RND efflux pump in *E. coli* as it is expressed constitutively under laboratory conditions and has been demonstrated to transport a wide range of substrates, including clinically relevant antimicrobials and host molecules such as bile salts [37, 38]. It was therefore unsurprising that *acrB* was more conserved than *acrF* across the pangenomes of 18 *E. coli* STs (1.2 alleles per pangenome for *acrB* compared with a mean of 1.7 for *acrF*) because despite the high nucleotide and amino acid similarity [15], AcrF has a much narrower substrate range than AcrB and unlike *acrB*, the *acrF* operon is HNS silenced [15, 18].

The absence of AcrF from the core genome of phylogroup E, the phylogenetic group to which ST11 (and therefore O157:H7) belongs, has been previously noted [39]. We believe the absence of this gene in both our work and a previously published pangenome study is due to the highly conserved insertion of 45 nucleotides, which when translated encodes 13 amino acids and two stop codons, thus resulting in two truncated products.

The loss of RND pumps has been linked to virulence attenuation in many Gram-negative bacteria including *Neisseria gonorrhoeae, E. amylovora* and *Vibrio cholerae* [13, 40, 41]. For example, *Salmonella* spp. possess a close homolog of AcrAB-TolC, and deletion of *acrB* in *Salmonella enterica* serovar Typhimurium has been shown to reduce pathogen colonization within the host [42]. Moreover, targeted inactivation of the RND pump AcrB via a single D408A point mutation has been demonstrated to result in decreased expression of *Salmonella* pathogenicity island genes, which facilitate bacterial invasion and replication within host epithelial cells [43]. Crucially, inactivation of *acrF* in *Salmonella* was shown to significantly reduce mortality in mice and reduce adhesion to and invasion of INT 407 cells [42, 44]. Given the well-documented virulence of ST11 *E. coli* within the human host, the lack of AcrF in this lineage is surprising and perhaps suggests that the importance of AcrF in virulence is species, or even lineage, dependent. It is possible this could be due to functional redundancy of RND efflux pumps in Gram-negative bacteria meaning the loss of AcrF function does not have a significant impact on the lifestyle of this *E. coli* lineage. Nevertheless, the findings presented here add to the growing notion that RND pump diversity and conservation is a key factor in the development of future antimicrobials and efflux pump inhibitors.

## Supplementary Data

Supplementary material 1
