# Supplementary material for: E. coli ST11 (O157:H7) does not encode a functional AcrF efflux pump
Source: Microbiology (Reading). 2023 Apr 19;169(4):001324. doi: 10.1099/mic.0.001324 (PMC10202319; doi:10.1099/mic.0.001324)
Supplement: Supplementary material 1 [file mic-169-1324-s001.pdf]

Supplementary figures and tables

Supplementary Table 1. Assemblies used in *acrF* alignment (Figure 1a)

Strain name	NCBI accession code	Reference in alignment
Escherichia coli O157:H7 str. EC4115	NC_011353.1	O157:H7 1
Escherichia coli O157:H7 str. TW14359	NC_013008.1	O157:H7 2
Escherichia coli O157:H7 str. SS17	NZ_CP008805.1	O157:H7 3
Escherichia coli O157:H7 str. SS52	NZ_CP010304.1	O157:H7 4
Escherichia coli O157:H7 str. Sakai	NC_002695.2	O157:H7 5
Escherichia coli O157:H7 str. EDL933	NZ_CP008957.1	O157:H7 6
Escherichia coli O157:H7 str. WS4202	NZ_CP012802.1	O157:H7 7
Escherichia coli O157:H7 str. FRIK2069	NZ_CP015846.1	O157:H7 8
Escherichia coli O157:H7 str. FRIK2533	NZ_CP015842.1	O157:H7 9
Escherichia coli O157:H7 str. FRIK2455	NZ_CP015843.2	O157:H7 10
Escherichia coli O157:H7 str. FRIK944	NZ_CP016625.1	O157:H7 11

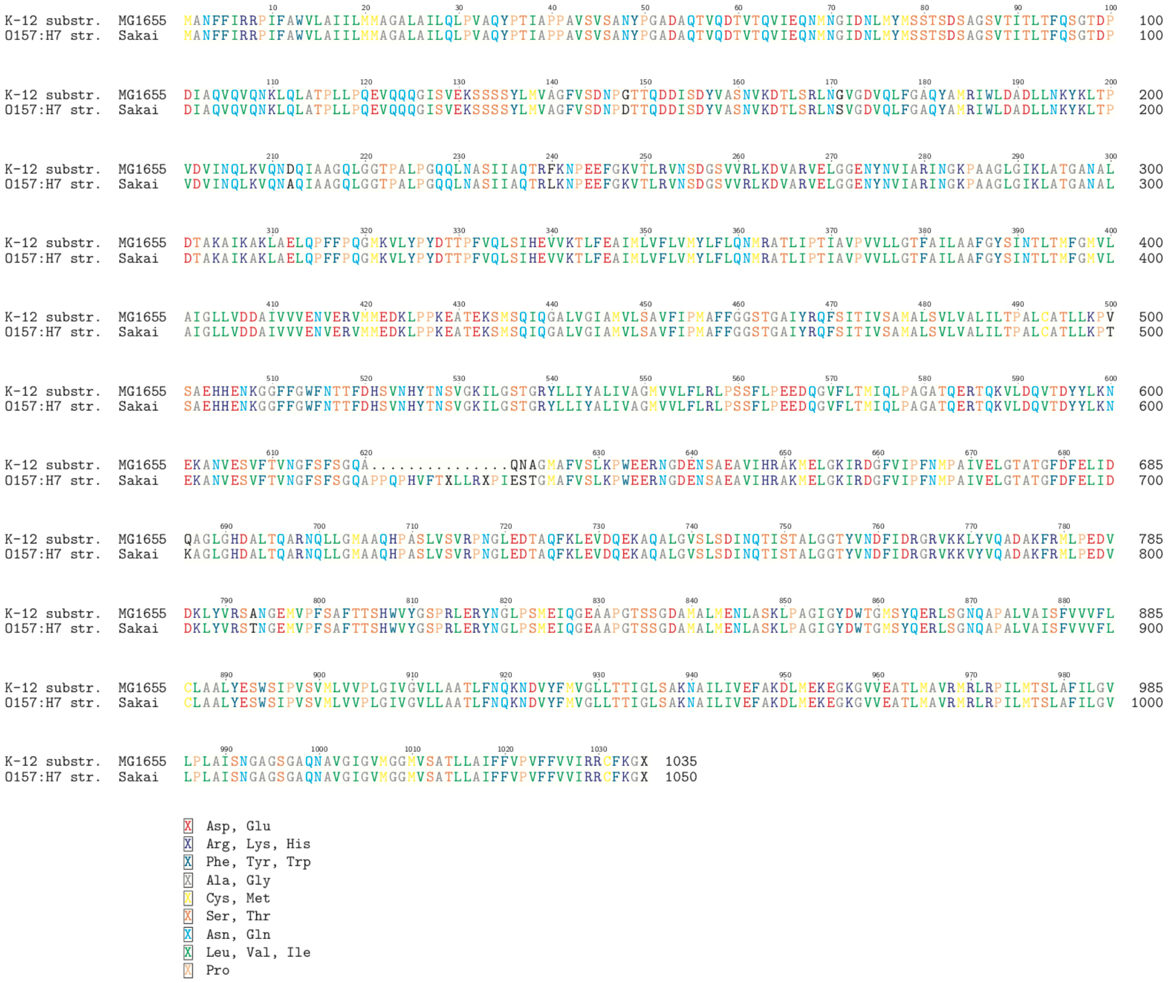

Supplementary Figure 1

Alignment of AcrF amino acid sequences from *E. coli* K-12 substr. MG1655 (accession: NC_000913.3) and *E. coli* O157:H7 str. Sakai (accession: NC_002695.2). Figure was generated using the MSA R package (1).

Reference

1. Bodenhofer U, Bonatesta E, Horejs-Kainrath C, Hochreiter S. msa: an R package for multiple sequence alignment. Bioinformatics. 2015;31(24):3997-9.
